# Ensemble machine learning algorithm for predicting acute kidney injury in patients admitted to the neurointensive care unit following brain surgery

**DOI:** 10.1038/s41598-023-33930-5

**Published:** 2023-04-25

**Authors:** Muying Wu, Xuandong Jiang, Kailei Du, Yingting Xu, Weimin Zhang

**Affiliations:** Intensive Care Unit, Affiliated Dongyang Hospital of Wenzhou Medical University, No. 60 Wuning West Road, Jinhua, Dongyang, Zhejiang People’s Republic of China

**Keywords:** Neurological disorders, Risk factors, Acute kidney injury

## Abstract

Acute kidney injury (AKI) is a common postoperative complication among patients in the neurological intensive care unit (NICU), often resulting in poor prognosis and high mortality. In this retrospective cohort study, we established a model for predicting AKI following brain surgery based on an ensemble machine learning algorithm using data from 582 postoperative patients admitted to the NICU at the Dongyang People's Hospital from March 1, 2017, to January 31, 2020. Demographic, clinical, and intraoperative data were collected. Four machine learning algorithms (C5.0, support vector machine, Bayes, and XGBoost) were used to develop the ensemble algorithm. The AKI incidence in critically ill patients after brain surgery was 20.8%. Intraoperative blood pressure; postoperative oxygenation index; oxygen saturation; and creatinine, albumin, urea, and calcium levels were associated with the postoperative AKI occurrence. The area under the curve value for the ensembled model was 0.85. The accuracy, precision, specificity, recall, and balanced accuracy values were 0.81, 0.86, 0.44, 0.91, and 0.68, respectively, indicating good predictive ability. Ultimately, the models using perioperative variables exhibited good discriminatory ability for early prediction of postoperative AKI risk in patients admitted to the NICU. Thus, the ensemble machine learning algorithm may be a valuable tool for forecasting AKI.

## Introduction

Acute kidney injury (AKI) is a common complication among neurocritical patients. According to the Kidney Disease: Improving Global Outcomes (KDIGO) study, the reported incidence of AKI is 11.6% in neurocritical patients, with approximately 3–12% of patients requiring hemodialysis^1^. Postoperative AKI is a major cause of poor outcomes after brain surgery. Many studies^2–4^ have confirmed that the occurrence of AKI is directly related to patient outcomes and results in a longer duration of postoperative mechanical ventilation, prolongation of the intensive care unit (ICU) and overall hospital stays, increased severity of disability, and significant increases in hospital mortality. The clinical diagnosis of AKI is largely dependent on serum creatinine (sCr) and urine output^5^. However, an increase in sCr usually indicates severely impaired renal function, and neurocritical patients often develop pituitary disorders, such as diabetes insipidus, leading to obvious abnormalities in urine output and consequently resulting in the inability to accurately diagnose AKI. Therefore, much research effort has been focused on the early and accurate identification of AKI in high-risk patients^6^.

Artificial intelligence (AI) and machine learning (ML) are regarded as highly valuable tools that can assist in preoperative planning, intraoperative guidance, and outcome prediction for patients undergoing neurosurgery^7^. Considerable research^8–10^ has focused on the application of ML methods in the prediction of postoperative mortality, recurrence, and adverse events in patients with brain tumors, neurovascular diseases, and traumatic brain injury. In recent years, ML has also been widely applied for the prediction of AKI in critically ill patients. For instance, He et al. extracted 76,957 encounters and relevant clinical variables from the electronic medical record system of a hospital to establish models for predicting AKI in general hospital populations using five ML methods^11^. Their results indicated that the constructed models provided good predictive performance. Although AI-based ML algorithms have shown promise for predicting AKI risk in several studies, the predictive performance of such models must be improved prior to widespread clinical application. Ensemble modeling, a relatively new approach that combines multiple ML models to enhance model accuracy, may be of value given recent evidence across medical specialties^12–14^.

Currently, there are few universal models for predicting the risk of AKI after brain surgery, making it difficult to utilize the large amounts of data available in modern electronic health record systems. Therefore, this study aimed to establish an ensemble learning-based predictive model using a large number of preoperative, intraoperative, and postoperative ICU variables, including intraoperative vital signs, medication information, and intake and output data. Improved accuracy in postoperative AKI prediction may aid in early evaluation of the patient’s risk level, assist in clinical decision-making, and promote timely intervention.

## Methods

### Study design and research participants

Our study was reported in accordance with the Transparent Reporting of a multivariable prediction model for Individual Prognosis or Diagnosis (TRIPOD) statement^15^ (Supplementary Table S1). This study was conducted in accordance with the tenets of the Declaration of Helsinki. Data were retrospectively analyzed for 582 postsurgical patients admitted to the ICU at the Dongyang People’s Hospital between March 1, 2017, and January 31, 2020. The inclusion criteria were as follows: (1) age ≥ 18 years and (2) ICU admission after brain surgery. The exclusion criteria were as follows: (1) chronic kidney disease, (2) AKI prior to surgery, (3) length of ICU stay < 24 h, and (4) percentage of missing data > 20%.

### Data collection

Data were collected using a medical record information mining software program provided by Shanghai LeJiu Healthcare Technology Co., Ltd (Shanghai, China). The collected data included the following: (1) demographic data: age, sex, diagnosis, comorbidities; (2) surgical data: operative time, type of surgery, intraoperative intake and output, intraoperative urine output, intraoperative vital signs (initial, maximum, minimum, and mean values), intraoperative blood transfusion data, intraoperative medication data; (3) blood gas and biochemical indicators 24 h after ICU admission; (4) use of drugs: commonly used diuretics, including mannitol, furosemide, and torasemide, and contrast media; and (5) clinical outcomes, including hospital mortality, duration of mechanical ventilation, and length of ICU stay.

This study was conducted in accordance with all related local guidelines and regulations, including those related to human genetics. This study was approved by the Ethics Committee of the Dongyang People’s Hospital (Dong Ren Yi 2021-YX-021), which waived the requirement for informed consent due to the retrospective and observational study design. The data were anonymized before analysis.

### Diagnostic criteria

AKI was defined and staged in accordance with the 2012 KDIGO criteria^16^: increases in sCr ≥ 50% or an sCr increase of at least 26.5 mol/L within 2 days. Chronic kidney disease was defined as a baseline estimated glomerular filtration rate < 60 mL/min/1.73 m^2^. The diagnosis of AKI was based solely on the serum creatinine levels according to the KDIGO criteria. Because of the retrospective nature of the study, we did not have accurate data on urine output. Baseline sCr was defined as the lowest sCr level within 6 months before ICU admission^17^. For patients without previous sCr data, it was estimated using the following formula^18^: sCr = 0.74–0.2 (if female) + 0.08 (if black) + 0.0039 × age (in years).

### Data processing

#### Selection of independent variables

A total of 119 variables were preliminarily selected based on their potential to influence AKI as demonstrated in the literature and clinical practice. First, variables with missing data > 20% were eliminated, after which 93 variables remained. Data preprocessing was then performed using the caret package in R language^19^ (R Software for Statistical Computing, Vienna, Austria) to remove variables strongly correlated with other variables, after which 80 variables remained. Subsequently, the backward feature selection method, random forest sampling, and tenfold cross-validation were adopted. After variable performance (error, forecast accuracy) had been calculated and the variables had been ranked in order of importance, the 10 most important variables were ultimately retained.

Outliers were detected using the interquartile range (IQR) (i.e., the difference between the upper and lower quartiles of the boxplot). The outliers were excluded and treated as missing values. Variables with > 20% missing values were deleted. The missing values of the variables were replaced using multiple imputations.

### Model construction

Four different models were first constructed using the C5.0, support vector machine (SVM), Bayes, and extreme gradient boosting (XGBoost) ML algorithms. Hyperparameter tuning was performed using the grid search method (Supplementary Figs. S1–3). Then, the four models were ensembled using the caretEnsemble package^14^ in R software. The resultant ensemble model was mainly trained using the following R packages: CARET, Ranger, arm, e1071, naivebayes, and gbm. The samples were randomly assigned to the training dataset for model training and test dataset for model validation in a 7:3 ratio. Supplementary Table S2 shows the comparison of baseline data and outcomes between the two datasets. All ML models were evaluated via tenfold cross-validation.

### Model validation and evaluation

The performance of each model was evaluated using the area under the receiver operating characteristic curve (AUC). The calibration performance of each model was evaluated using calibration curves. The confusion matrix was evaluated based on accuracy, precision, specificity, recall, and balanced accuracy using a cut-off of 0.5.

Models were interpreted based on variable importance, which was sorted using the function “varImp” within the CARET package in R. A locally weighted regression technique was used to detect the cut-off values of the top four features that predicted AKI. Moreover, iBreakdown algorithms were used for individual interpretation^20^.

### Statistical analysis

Descriptive statistical analyses were performed using the CBCgrps package in R^21^. Normally distributed measurement data were expressed as x ± s and compared between the groups using two-independent-samples *t*-tests. Meanwhile, non-normally distributed data were expressed as medians (P25, P75) and compared using the Mann–Whitney U tests. Enumeration data were expressed in terms of the rate and percentage and compared between groups using the χ^2^ test. All statistical analyses were performed using R software version 4.1.2. A P-value < 0.05 was considered significant.

### Approval for human experiments

This study was approved by the Ethics Committee of Dongyang People’s Hospital (Dong Ren Yi 2021-YX-021).

### Informed consent

The requirement for informed consent was waived due to the retrospective and observational study design.

## Results

### Comparison of baseline data

**Figure 1:**
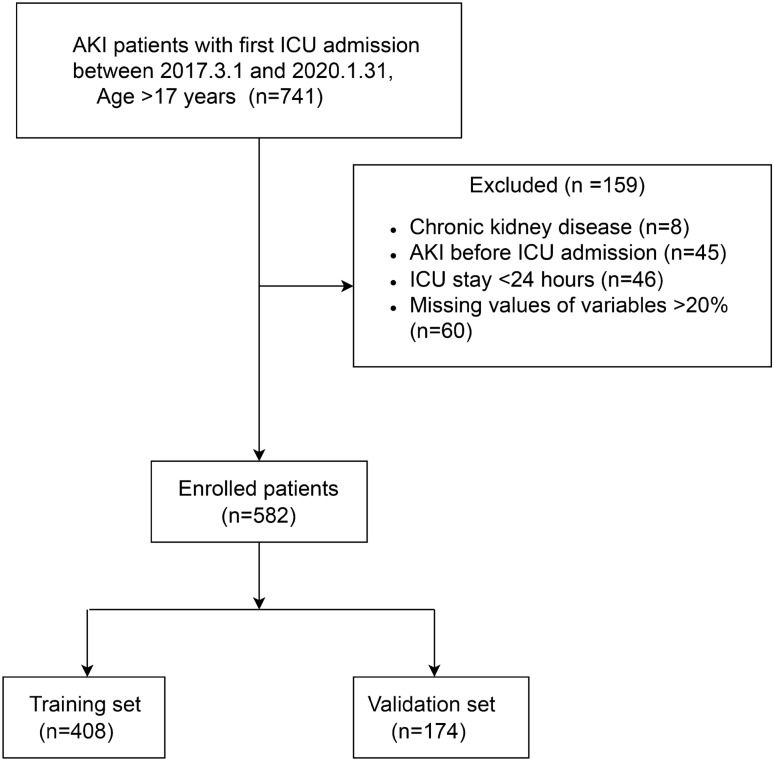
Flow chart of the study. ICU, Intensive Care Unit; AKI, acute kidney injury.

A total of 582 postsurgical neurocritical patients were included in this study. Figure 1 shows the flowchart of the participant selection process. Among the included patients, the incidence of AKI was 20.8% (121 patients). The numbers of patients with Stage I, II, and III AKI were 108 (89.2%), three (2.5%), and 10 (8.3%), respectively. Table 1 shows the comparison of baseline clinical data and clinical outcomes between the AKI and non-AKI groups. Age and sex did not significantly differ between the groups, with the mean age and proportion of men among patients being 56.4 ± 15.2 years and 58%, respectively. The AKI group had a significantly higher mean APACHE score. Overall, 112 of the 582 patients died (overall hospital mortality: 19.2%). While mortality was higher in the AKI group than in the non-AKI group, the difference was not statistically significant (25% vs 18%, P = 0.082). Significant differences were observed for the duration of mechanical ventilation, length of ICU stay, length of hospital stay, and total cost of hospitalization (P < 0.001), with outcomes being poorer in the AKI group.

**Table 1:** Comparisons of baseline characteristics and outcomes between patients with and without AKI.

Variables	No AKI (n = 461)	AKI (n = 121)	P-value
Age (years), mean (SD)	56 ± 15	58 ± 15	0.2
Male, n (%)	264 (57%)	74 (61%)	0.4
Smoking, n (%)	147 (32%)	53 (44%)	0.014
Alcohol drinking, n (%)	176 (38%)	57 (47%)	0.074
APACHE II (score), mean (SD)	19 ± 6	21 ± 5	< 0.001
Contrast media use, n (%)	175 (38%)	34 (28.1)	0.044
Diuretic use, n (%)	108 (23%)	52 (43%)	< 0.001
Comorbidities, n (%)			
Hypertension	191 (41%)	62 (51%)	0.053
Diabetes	26 (5.6%)	11 (9.1%)	0.2
Tumor	17 (3.7%)	7 (5.8%)	0.3
Laboratory indexes			
Creatinine (mmol/L)	57 ± 16	80 ± 19	< 0.001
Urea (mmol/L), median (IQR)	4.26 (3.36, 5.33)	5.43 (4.12, 7.36)	< 0.001
White blood cell (× 109/L), mean (SD)	11.2 ± 3.8	11.7 ± 3.8	0.2
Red blood cell(× 109/L), mean (SD)	3.64 ± 0.55	3.66 ± 0.63	0.4
Platelet count (× 109/L), mean (SD)	165 ± 56	152 ± 60	0.021
pH, mean (SD)	7.41 ± 0.06	7.40 ± 0.06	0.3
Bicarbonate (mmol/L), mean (SD)	22.8 ± 2.8	23.2 ± 2.9	0.3
Lactate (mmol/L), median (IQR)	2.10 (1.40, 2.90)	2.30 (1.50, 3.00)	0.12
Oxyhemoglobin saturation (%), median (IQR)	99.10 (98.00, 99.60)	98.30 (96.60, 99.40)	< 0.001
Calcium (mmol/L), mean (SD)	2.04 ± 0.14	2.00 ± 0.16	0.019
Albumin (g/L), mean (SD)	34.0 ± 4.1	31.9 ± 4.3	< 0.001
Outcome			
ICU length of stay (days), median (IQR)	5 (2, 10)	12 (6, 20)	< 0.001
Ventilation duration (days), median (IQR)	2.2 (0.7, 5.8)	6.5 (2.1, 12.3)	< 0.001
Hosp. LOS (days), median (IQR)	18 (13, 23)	21 (14, 29)	0.001
Cost (× 103 yuan), median (IQR)	55.4 (43.3, 73.4)	73.1 (53.4, 97.8)	< 0.001
Hospital mortality, n (%)	82 (18%)	30 (25%)	0.082

AKI, acute kidney injury; APACHE, Acute Physiology and Chronic Health Evaluation; ICU, intensive care unit; Hosp. LOS, length of hospital stay.

### Comparison of surgical data

Among the 582 postsurgical patients included in this study, 517 (87.4%) underwent emergency surgery. Surgery was performed in the subdural space in 168 patients (28.9%), epidural space in 39 patients (6.7%), supratentorial lesions in 178 patients (30.6%), cranial base and posterior cranial fossa in 23 patients (3.9%), and other sites in 174 patients (29.9%). Table 2 shows the comparison of intraoperative data between the AKI and non-AKI groups.

**Table 2:** Comparisons of intraoperative data between patients with and without AKI.

Variables	No AKI (n = 461)	AKI (n = 121)	P-value
Time of operation (hours), mean (SD)	3.36 ± 1.2	3.12 ± 1.44	0.2
Bleeding (mL), median (IQR)	300 (100, 400)	300 (200, 400)	0.3
Fluid intake (mL), median (IQR)	2,559 (2,055, 3,053)	2,557 (2,070, 3,339)	0.3
Fluid output (mL), median (IQR)	900 (500, 1,300)	900 (500, 1,500)	0.4
Urine (mL), median (IQR)	500 (300, 1,000)	500 (300, 900)	> 0.9
Intraoperative vital signs, mean (SD)			
Minimum systolic pressure (mmHg)	99 ± 11	97 ± 17	> 0.9
Maximum systolic pressure (mmHg)	156 ± 19	161 ± 21	0.005
Average systolic pressure (mmHg)	123 ± 10	125 ± 12	0.022
Minimum diastolic pressure (mmHg)	54 ± 8	54 ± 10	> 0.9
Maximum diastolic pressure (mmHg)	86 ± 12	89 ± 14	0.013
Average diastolic pressure (mmHg)	67 ± 8	68 ± 9	0.2
Initial Mean diastolic pressure (mmHg)	93 ± 11	94 ± 11	0.4
Initial Temperature (°C)	36.68 ± 0.58	36.69 ± 0.61	> 0.9
Initial Heart rate (bpm)	82 ± 17	87 ± 18	0.004
Transfusion			
RBC transfusion, n (%)	86 (19%)	29 (24%)	0.2
FFP transfusion, n (%)	87 (19%)	32 (26%)	0.066
Platelet transfusion, n (%)	18 (3.9%)	12 (9.9%)	0.008

AKI, acute kidney injury; RBC, red blood cells; FFP, fresh frozen plasma.

### Evaluation of ML models

**Figure 2:**
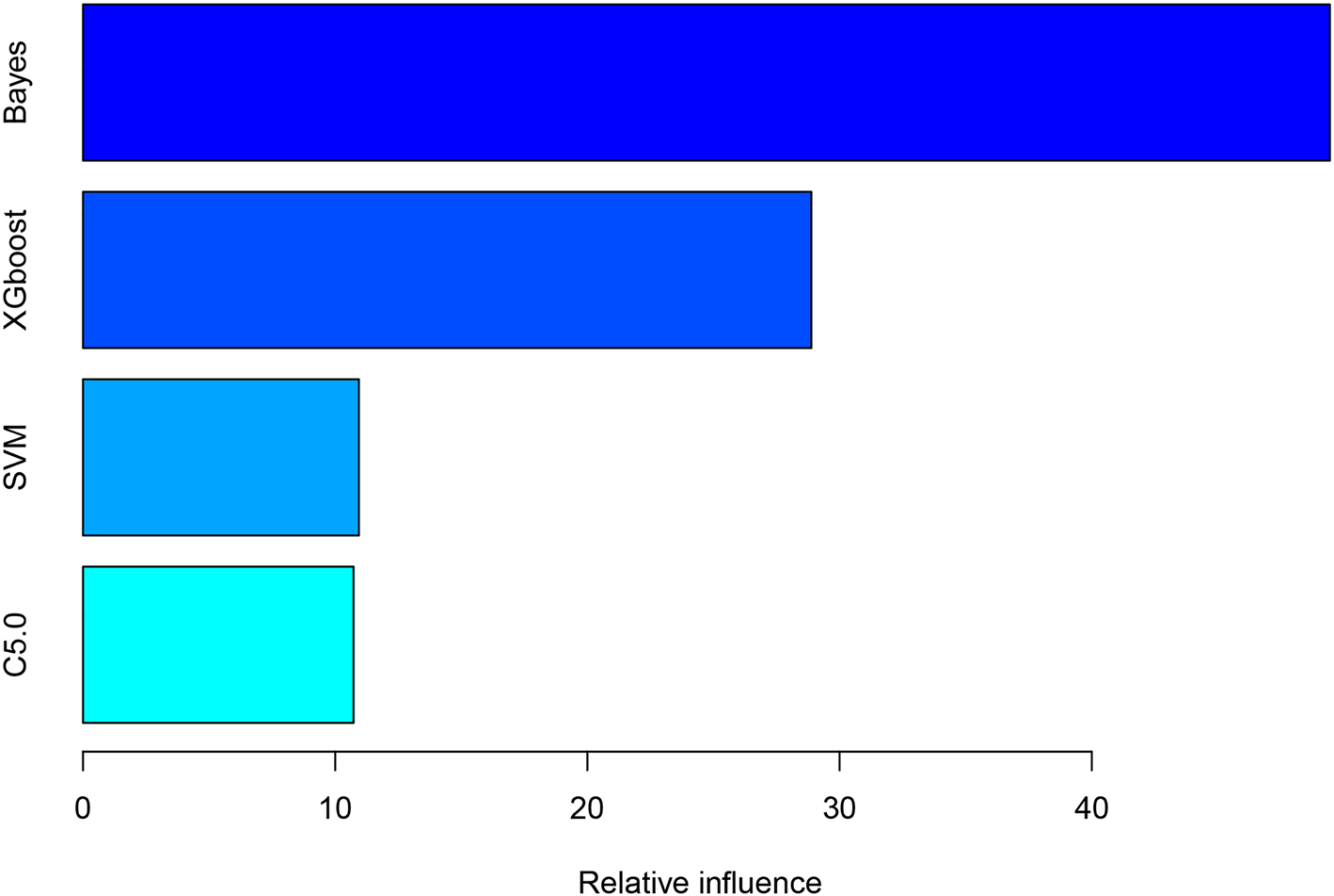
Relative influence of each machine learning model in the stacked-ensemble model. SVM, support vector machine; XGBoost, extreme gradient boosting.

**Figure 3:**
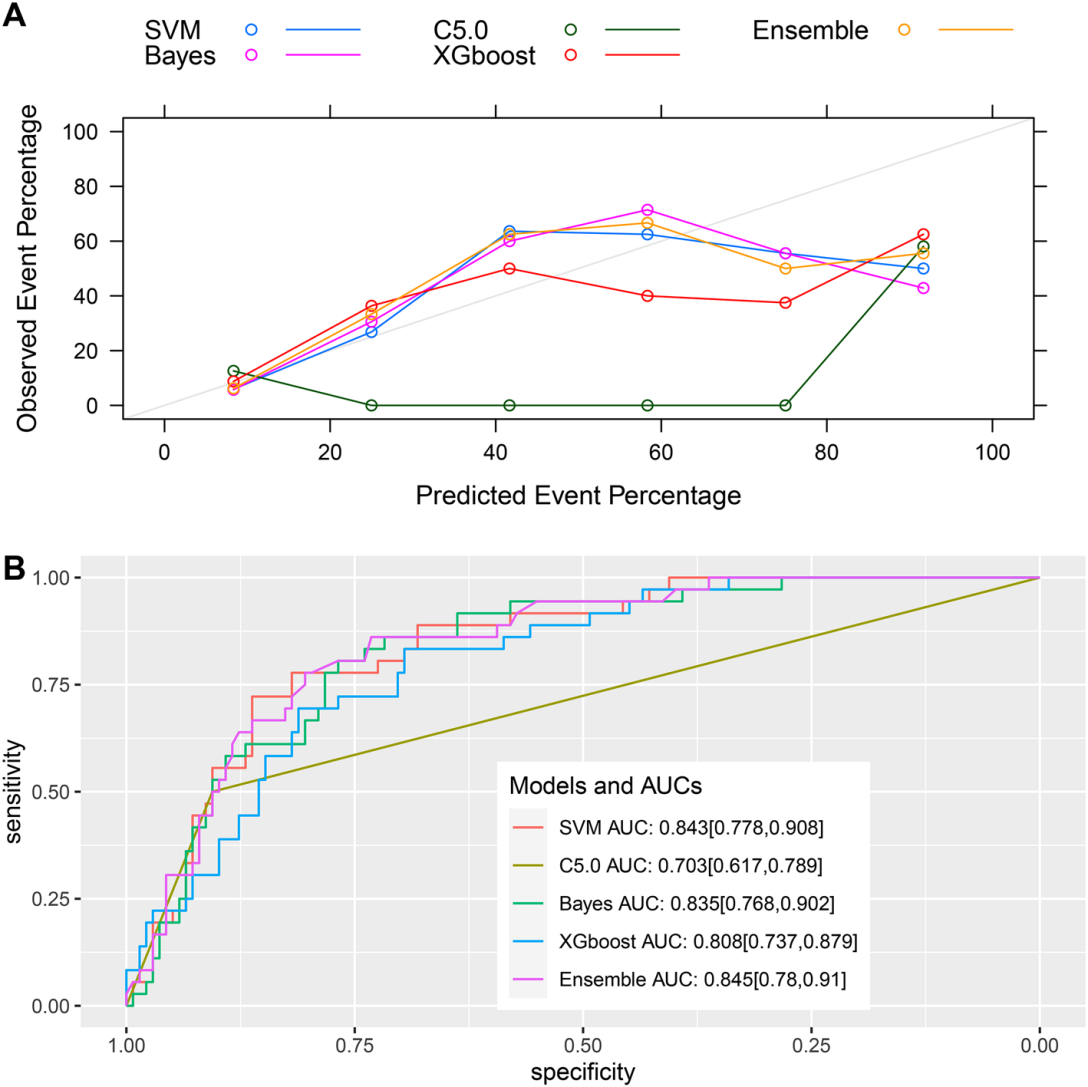
Evaluation of model performance in the internal validation dataset. (**A**) The calibration plot shows the consistency between observed and predicted risks for persistent acute kidney injury. (**B**) Discrimination of the machine learning models in the internal validation dataset. SVM, support vector machine; XGBoost, extreme gradient boosting; AUC, area under the curve. The number in parentheses indicates the 95% confidence interval.

ML models were established using the C5.0, SVM, Bayes, and XGBoost ML algorithms and subsequently ensembled. Figure 2 shows the relative influences of the individual ML models on the ensemble model, demonstrating that the Bayes and XGBoost models exerted the greatest influence. Figure 3 shows the receiver operating characteristic curves and calibration plots for the five different predictive models for the validation dataset. The AUC value was greatest for the ensemble model (0.85, 95% CI 0.78–0.91) followed by the SVM model (0.84, 95% CI 0.78–0.91). All models, except C5.0, exhibited good predictive performance. Table 3 shows the confusion matrix for the predictive models. The accuracy, precision, specificity, recall and balanced accuracy values of the ensemble model were 0.81, 0.86, 0.44, 0.91, and 0.68, respectively.

**Table 3:** Comparison of machine learning model performance in the internal validation set.

	Models for predicting persistent AKI in internal validation				
	SVM	C5.0	XGBoost	Bayes	Ensemble
Accuracy (95% CI)	0.81 (0.74, 0.87)	0.82 (0.76, 0.88)	0.78 (0.71, 0.84)	0.81 (0.74, 0.87)	0.81 (0.74, 0.87)
Precision	0.84	0.87	0.84	0.85	0.86
Recall	0.93	0.90	0.90	0.93	0.91
Specificity	0.33	0.50	0.33	0.36	0.44
Balanced accuracy	0.63	0.70	0.62	0.64	0.68

AKI: acute kidney injury; SVM = support vector machine; XGBoost = extreme gradient boosting; CI = confidence interval.

**Figure 4:**
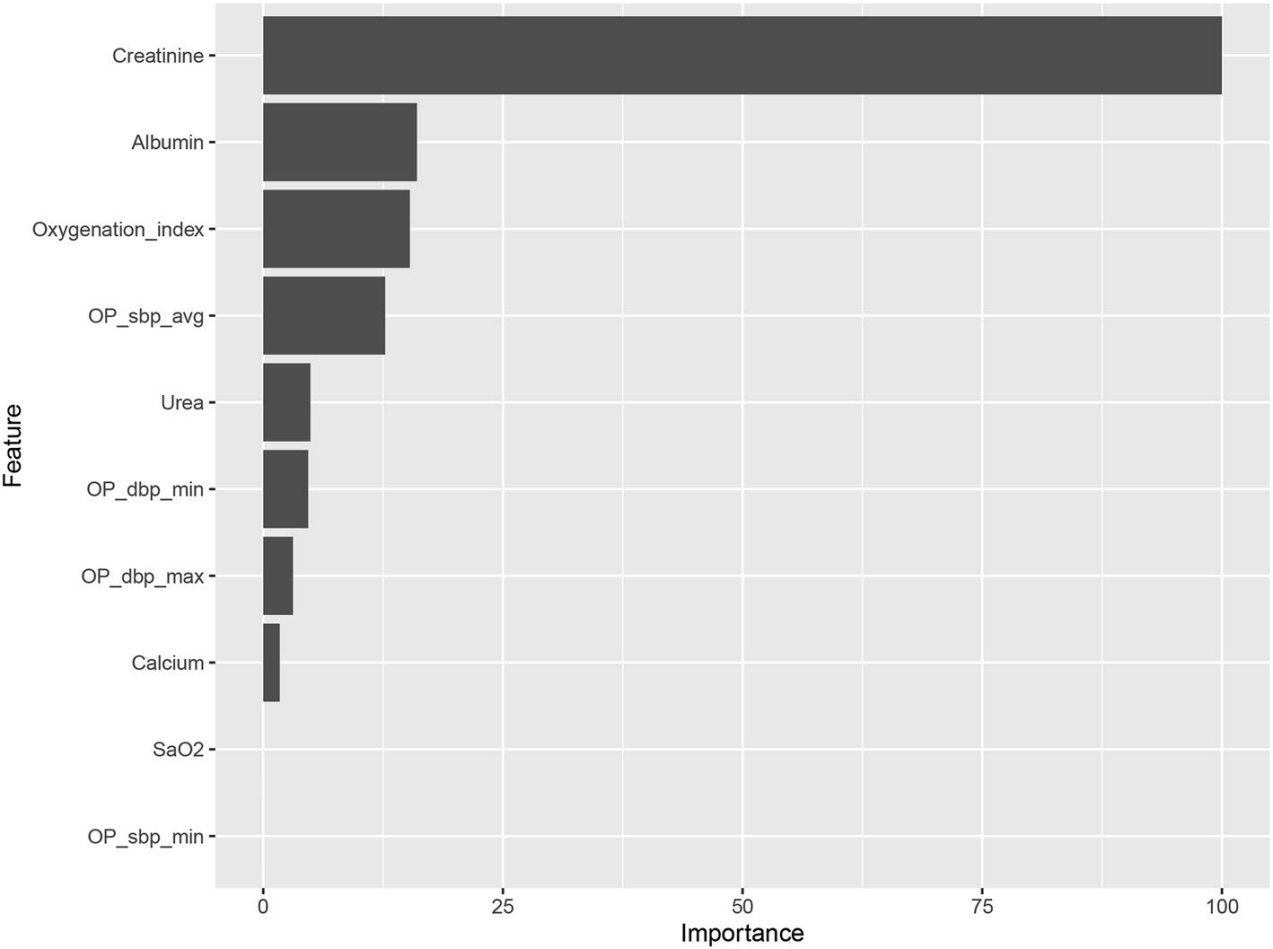
Variable-importance ranking in the ensemble model. SaO2, Oxyhemoglobin saturation; OP_sbp_avg, average systolic pressure; OP_dbp_min, Minimum diastolic pressure; OP_dbp_max, Maximum diastolic pressure; OP_sbp_min, Minimum systolic pressure.

**Figure 5:**
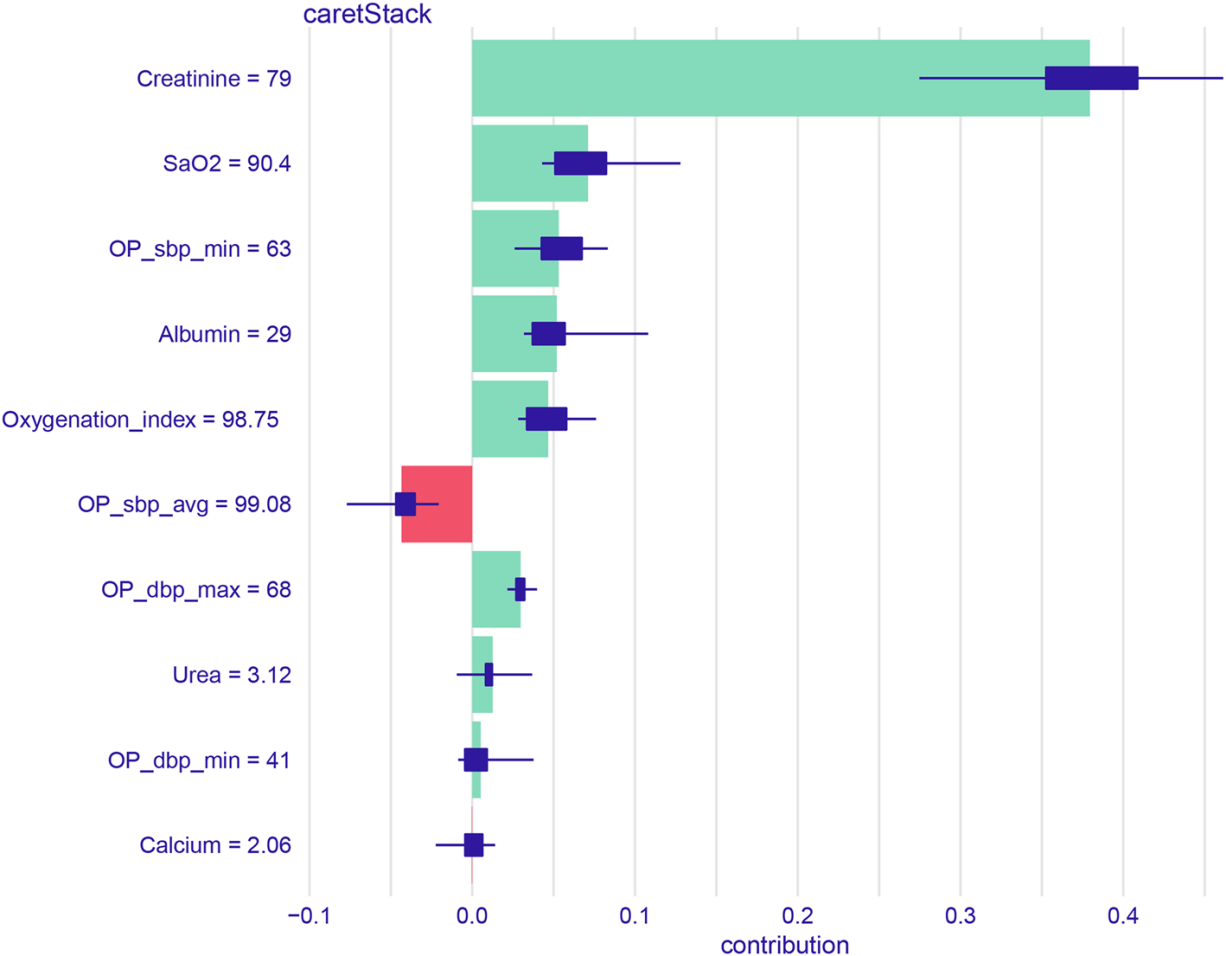
Model interpretation using the iBreakdown algorithm with uncertainty indicated by a box plot. The horizontal axis reflects the probability scale. SaO_2_, Oxyhemoglobin saturation; OP_sbp_avg, average systolic pressure; OP_dbp_min, Minimum diastolic pressure; OP_dbp_max, Maximum diastolic pressure; OP_sbp_min, Minimum systolic pressure.

Supplementary Figure S4 shows the crude relationships between the top four features and AKI. Figure 4 and Supplementary Fig. S5 show the order of importance for variables in the five ML models. The creatinine level, oxygenation index, and albumin level on ICU admission and the intraoperative mean systolic blood pressure exerted the greatest influence on the models. Figure 5 shows the iBreakdown results regarding the contribution of each variable to the probability of AKI for Patient 1. The analysis indicated that an initial sCr = 79 was closely correlated with a reduced risk of AKI.

## Discussion

In the present study, approximately one in five patients admitted to the ICU following brain surgery developed AKI, which is consistent with previous results^22^. Given the availability of considerable amounts of perioperative data, we adopted four commonly used ML methods to develop models for the early prediction of postoperative AKI. When the four models were ensembled, the resultant model exhibited better predictive ability, achieving an AUC of approximately 0.85, as well as satisfactory values for accuracy and recall.

C5.0, SVM, Bayes, and XGBoost are the most common ML algorithms. The Bayes method provides the basis for probability learning methods, while the SVM is commonly used for classification. Both C5.0 and XGBoost are classic ML algorithms. These algorithms have good prediction effect for binary classification problems. However, the order of importance for variables differed for the five ML models. For example, the creatinine level on ICU admission played a major role in the C5.0 model. In the confusion matrix, the C5.0 model exhibited the best performance, with the highest accuracy, precision, specificity, and balanced accuracy values. However, the AUC value for the C5.0 model had poor performance and the worst calibration. The accuracy values for the SVM, Bayes, and ensemble models were similar, although the balanced accuracy and specificity values were higher for the ensemble model.

Similar to the present study, Adhikari et al.^23^ utilized intraoperative physiologic data, laboratory indicators, and medication information from 2,911 adult surgical patients to develop a model for predicting postoperative AKI, which exhibited an AUC of 0.86. The authors also noted that incorporating intraoperative physiologic time series data enabled better identification of patients at risk for AKI. Lei et al.^24^ adopted ML algorithms for the prediction of postoperative AKI using data from 42,615 patients who underwent major non-cardiac surgery. Their results indicated that the inclusion of preoperative and intraoperative data increased the AUC of the gradient boosting machine from 0.712 to 0.804. Our results support the notion that ensemble models based on perioperative data can promote early and accurate prediction of post-brain surgery AKI.

In the present study, the following variables were ranked highest in importance among the five ML models: initial creatinine level, urea level on ICU admission, intraoperative blood pressure, postoperative oxygenation index, and serum albumin level. Although the cause–effect relationships between each of these factors and postoperative AKI cannot be elucidated owing to the black-box nature of ML algorithms, the presence of significant correlations is certain. Therefore, we used a locally weighted scatterplot smoothing technique to assess the crude relationships between the top four features and AKI. The postoperative oxygenation index was negatively associated with AKI, with lower oxygenation values corresponding to higher AKI risk. The analysis indicated that the risk of AKI was lowest at a creatinine level of 40 μmoI/L, an albumin level of 39 mmol/l, and an average systolic pressure of 116 mmHg. Common causes of AKI include infection, hypovolemia, and hypoperfusion, which tend to cause hypoxia. Therefore, hypoxia is regarded as a frequent pathophysiological manifestation of AKI^25^. Arterial blood pressure is also closely related to AKI following high-risk surgery^26^. In a previous study, predictive models for postoperative AKI in the ICU were established using preoperative variables and intraoperative blood pressure^27^, yielding good predictive effects. Other studies have reported a close relationship between serum albumin level and the occurrence of postoperative AKI^28,29^. For instance, one retrospective cohort study reported that a low serum albumin level immediately after major abdominal surgery increased the risk of postoperative AKI^30^. Therefore, monitoring and control of intraoperative blood pressure, postoperative oxygenation index, and serum albumin level may aid in preventing AKI and provide guidance for effective clinical treatment.

The present study has certain limitations. First, this was a single-center retrospective study, in which samples were randomly divided into the training and test datasets in a 7:3 ratio. The lack of validation using external data therefore limits the applicability of the model results. Second, the ML algorithms used in this study may exhibit poor interpretability. To address this issue, the variables included in the models were ranked in the order of importance, and the iBreakdown algorithm was used for direct interpretations in individual patients. Third, given that the ratio between patients with and without AKI was 1:4, the dataset used for the present study was imbalance. However, we attempted to account for this using accuracy, precision, specificity, recall, and balanced accuracy values in addition to the common ROC model. Finally, the models constructed in the present study are applicable only for predicting the risk of AKI occurrence during the ICU stay. In future studies, the possibility of AKI occurring 24 or 48 h postoperatively should be investigated using preoperative and intraoperative variables to enhance the predictability and temporal validity of the models.

In conclusion, our study showed that AKI is a prevalent condition affecting neurocritical patients. Through utilizing a comprehensive suite of perioperative variables, we have successfully developed an ensemble machine learning model that exhibits excellent predictive ability in forecasting AKI following brain surgery. However, future prospective studies will be necessary to validate the results of this study.

## Supplementary Information

Supplementary Information.

## Data Availability

The data are available from the corresponding author on reasonable request. The codes are available at: https://github.com/fzs1412/Ensemble-Machine-Learning-Algorithm.git.

## References

[1] Ramírez-Guerrero G, Baghetti-Hernández R, Ronco C (2022). Acute kidney injury at the neurocritical care unit. Neurocrit. Care.

[2] Büttner S (2020). Incidence, risk factors, and outcome of acute kidney injury in neurocritical care. J. Intensive Care Med..

[3] Deng Y (2017). The incidence, risk factors and outcomes of postoperative acute kidney injury in neurosurgical critically ill patients. Sci. Rep..

[4] Kovacheva VP (2016). Acute kidney injury after craniotomy is associated with increased mortality: a cohort study. Neurosurgery.

[5] Chawla LS (2017). Acute kidney disease and renal recovery: Consensus report of the Acute Disease Quality Initiative (ADQI) 16 Workgroup. Nat. Rev. Nephrol..

[6] Zhang Z (2019). Machine learning method for the management of acute kidney injury: more than just treating biomarkers individually. Biomark. Med..

[7] Senders J (2018). An introduction and overview of machine learning in neurosurgical care. Acta Neurochir..

[8] Abujaber A (2020). Prediction of in-hospital mortality in patients on mechanical ventilation post traumatic brain injury: Machine learning approach. BMC Med. Inform. Decision Mak..

[9] Guo Y (2020). Distinguishing focal cortical dysplasia from glioneuronal tumors in patients with epilepsy by machine learning. Front. Neurol..

[10] He XW, Du CN, Zhao K, Yang MF, Ma QF (2020). A novel model for predicting the outcome of intracerebral hemorrhage: Based on 1186 patients. J. Stroke Cerebrovasc. Dis..

[11] He J, Hu Y, Zhang X, Wu L, Waitman LR, Liu M (2019). Multi-perspective predictive modeling for acute kidney injury in general hospital populations using electronic medical records. JAMIA Open..

[12] Muhlestein WE, Akagi DS, Davies JM, Chambless LB (2019). Predicting inpatient length of stay after brain tumor surgery: Developing machine learning ensembles to improve predictive performance. Neurosurgery.

[13] Bannick MS, McGaughey M, Flaxman AD (2021). Ensemble modelling in descriptive epidemiology: burden of disease estimation. Int. J. Epidemiol..

[14] Zhang Z, Chen L, Xu P, Hong Y (2022). Predictive analytics with ensemble modeling in laparoscopic surgery: a technical note. Laparoscop. Endoscop. Robotic Surg..

[15] Collins GS, Reitsma JB, Altman DG, Moons KG (2015). Transparent reporting of a multivariable prediction model for individual prognosis or diagnosis (TRIPOD): The TRIPOD statement. J. Brit. Surg..

[16] Khwaja A (2012). KDIGO Clinical practice guidelines for acute kidney injury. Nephron. Clin. Pract..

[17] Shen Y, Zhang W, Cheng X, Ying M (2018). Association between postoperative fluid balance and acute kidney injury in patients after cardiac surgery: A retrospective cohort study. J. Crit. Care..

[18] Závada J (2010). A comparison of three methods to estimate baseline creatinine for RIFLE classification. Nephrol. Dialys. Transpl..

[19] Kuhn, M. Caret: classification and regression training. Astrophysics Source Code Library, ascl-1505 (2015).

[20] Staniak M, Biecek P. Explanations of model predictions with live and breakDown packages. The R Foundation (2019).

[21] Zhang Z, Gayle AA, Wang J, Zhang H, Cardinal-Fernandez P (2017). Comparing baseline characteristics between groups: An introduction to the CBCgrps package. Ann. Transl. Med..

[22] Zorrilla-Vaca A (2018). Acute kidney injury following acute ischemic stroke and intracerebral hemorrhage: A meta-analysis of prevalence rate and mortality risk. Cerebrovasc. Dis..

[23] Adhikari L, Ozrazgat-Baslanti T, Ruppert M (2019). Improved predictive models for acute kidney injury with IDEA: Intraoperative data embedded analytics. PLoS ONE.

[24] Lei VJ (2019). Risk stratification for postoperative acute kidney injury in major noncardiac surgery using preoperative and intraoperative data. JAMA Netw. Open..

[25] Basile DP, Anderson MD, Sutton TA (2012). Pathophysiology of acute kidney injury. Comprehens. Physiol..

[26] Greco, M., Falini, S., Angelotti, G. & Cecconi, M. Association of mean arterial pressure and acute kidney injury after high risk surgery. Intensive Care Medicine Experimental Conference: 32nd European Society of Intensive Care Medicine Annual Congress, ESICM. 7 (2019).

[27] Bihorac A (2013). Database communication enables machine learning classifiers to predict postoperative AKI in ICU. Crit. Care Med..

[28] Thongprayoon C, Cheungpasitporn W, Mao MA, Sakhuja A, Kashani K (2018). U-shape association of serum albumin level and acute kidney injury risk in hospitalized patients. PLoS ONE.

[29] Shao M, Wang S, Parameswaran PK (2017). Hypoalbuminemia: a risk factor for acute kidney injury development and progression to chronic kidney disease in critically ill patients. Int. Urol. Nephrol..

[30] Li W, Li N, Li S (2021). Relationship between postoperative immediate serum albumin level and postoperative acute kidney injury after major abdominal surgery in critically ill patients. Zhonghua Wei Zhong Bing Ji Jiu Yi Xue.

