# Supplementary material for: Ensemble machine learning algorithm for predicting acute kidney injury in patients admitted to the neurointensive care unit following brain surgery
Source: Sci Rep. 2023 Apr 25;13:6705. doi: 10.1038/s41598-023-33930-5 (PMC10130041; doi:10.1038/s41598-023-33930-5)
Supplement: Supplementary file 1 — Supplementary Information. [file 41598_2023_33930_MOESM1_ESM.pdf]

Supplementary Information

Ensemble Machine Learning Algorithm for Predicting Acute Kidney Injury in Patients Admitted to the Neurointensive Care Unit following Brain Surgery

Muying Wu[1], Xuandong Jiang[1*✉], Kailei Du[1], Yingting Xu[1], Weimin Zhang[1]

[1] Intensive Care Unit, Affiliated Dongyang Hospital of Wenzhou Medical University, Dongyang, Zhejiang, P.R. China

***Corresponding author:** Xuandong Jiang, MD

Intensive Care Unit, Affiliated Dongyang Hospital of Wenzhou Medical University, Dongyang, Zhejiang, P.R. China, No. 60 Wuning West Road, Jinhua, China

Email: lxqjiang@hotmail.com

Tel: 86-571-87236838

Fax: 86-571-87236838

Table S1 Transparent Reporting of a multivariable prediction model for Individual Prognosis Or Diagnosis

**Section/Topic**			**Checklist Item**	**Page**
**Title and abstract**				
Title	1	D;V	Identify the study as developing and/or validating a multivariable prediction model, the target population, and the outcome to be predicted.	Title
Abstract	2	D;V	Provide a summary of objectives, study design, setting, participants, sample size, predictors, outcome, statistical analysis, results, and conclusions.	Abstract
				**Introduction**
Background and objectives	3a	D;V	Explain the medical context (including whether diagnostic or prognostic) and rationale for developing or validating the multivariable prediction model, including references to existing models.	Introduction
	3b	D;V	Specify the objectives, including whether the study describes the development or validation of the model or both.	Introduction
			**Methods**	
Source of data	4a	D;V	Describe the study design or source of data (e.g., randomized trial, cohort, or registry data), separately for the development and validation data sets, if applicable.	Methods, Research Participants
	4b	D;V	Specify the key study dates, including start of accrual; end of accrual; and, if applicable, end of follow-up.	Methods, Study Design
Participants	5a	D;V	Specify key elements of the study setting (e.g., primary care, secondary care, general population) including number and location of centres.	Methods, Study Design
	5b	D;V	Describe eligibility criteria for participants.	Methods, Research Participants
	5c	D;V	Give details of treatments received, if relevant.	Not applicable
Outcome	6a	D;V	Clearly define the outcome that is predicted by the prediction model, including how and when assessed.	Methods, Diagnostic criteria
	6b	D;V	Report any actions to blind assessment of the outcome to be predicted.	Not applicable
Predictors	7a	D;V	Clearly define all predictors used in developing the multivariable prediction model, including how and when they were measured.	Methods, Data collection
	7b	D;V	Report any actions to blind assessment of predictors for the outcome and other predictors.	Not applicable
Sample size	8	D;V	Explain how the study size was arrived at.	Not applicable
Missing data	9	D;V	Describe how missing data were handled (e.g., complete-case analysis, single imputation, multiple imputation) with details of any imputation method.	Methods, Data Processing
Statistical analysis methods	10a	D	Describe how predictors were handled in the analyses.	Methods, Statistical analysis
	10b	D	Specify type of model, all model-building procedures (including any predictor selection), and method for internal validation.	Methods, Model construction
	10c	V	For validation, describe how the predictions were calculated.	Methods, Model evaluation
	10d	D;V	Specify all measures used to assess model performance and, if relevant, to compare	Methods,

			multiple models.	Model evaluation
	10e	V	Describe any model updating (e.g., recalibration) arising from the validation, if done.	Not applicable
Risk groups	11	D;V	Provide details on how risk groups were created, if done.	Not applicable
Development vs. validation	12	V	For validation, identify any differences from the development data in setting, eligibility criteria, outcome, and predictors.	Methods, Model validation
Results				
Participants	13a	D;V	Describe the flow of participants through the study, including the number of participants with and without the outcome and, if applicable, a summary of the follow-up time. A diagram may be helpful.	Results, Figure 1
	13b	D;V	Describe the characteristics of the participants (basic demographics, clinical features, available predictors), including the number of participants with missing data for predictors and outcome.	Results, Table 2-3
	13c	V	For validation, show a comparison with the development data of the distribution of important variables (demographics, predictors and outcome).	Results, Table S2
Model development	14a	D	Specify the number of participants and outcome events in each analysis.	Results
	14b	D	If done, report the unadjusted association between each candidate predictor and outcome.	Not applicable
Model specification	15a	D	Present the full prediction model to allow predictions for individuals (i.e., all regression coefficients, and model intercept or baseline survival at a given time point).	Results, Model interpretation
	15b	D	Explain how to use the prediction model.	Results, Figure 5
Model performance	16	D;V	Report performance measures (with CIs) for the prediction model.	Results, Table 3
Model-updating	17	V	If done, report the results from any model updating (i.e., model specification, model performance).	Not applicable
**Discussion**				
Limitations	18	D;V	Discuss any limitations of the study (such as nonrepresentative sample, few events per predictor, missing data).	Discussion Limitations
Interpretation	19a	V	For validation, discuss the results with reference to performance in the development data, and any other validation data.	Discussion
	19b	D;V	Give an overall interpretation of the results, considering objectives, limitations, results from similar studies, and other relevant evidence.	Discussion, Conclusion
Implications	20	D;V	Discuss the potential clinical use of the model and implications for future research.	Conclusion
**Other information**				
Additional information	21	D;V	Provide information about the availability of Additional resources, such as study protocol, Web calculator, and data sets.	Not applicable
Funding	22	D;V	Give the source of funding and the role of the funders for the present study.	Declarations Funding

*Items relevant only to the development of a prediction model are denoted by D, items relating solely to a validation of a prediction model are denoted by V, and items relating to both are denoted D;V. We recommend using the TRIPOD Checklist in conjunction with the TRIPOD Explanation and Elaboration document.

Table S2. Comparison of feature distributions and outcomes between the training and validation groups

**Variables**	Training group (n = 683)	Validation group (n = 292)	P-value
Age (years), mean (SD)	57 ±15	54 ±16	0.020
Male, n (%)	229 (56%)	109 (63%)	0.14
APACHE (score), mean (SD)	19 ±6	19 ±6	0.9
AKI stages, n (%)			0.9
0	323 (79%)	138 (79%)	
1	77 (19%)	31 (18%)	
2	2 (0.5%)	1 (0.6%)	
3	6 (1.5%)	4 (2.3%)	
RRT, n (%)	4 (1.0%)	0 (0%)	0.3
Intraoperative data			
Time of operation (hours), mean (SD)	3.12 ±1.20	3.36 ±1.20	0.12
Minimum diastolic pressure (mmHg), mean (SD)	54 ±9	54 ±8	0.5
Maximum diastolic pressure (mmHg), mean (SD)	87 ±13	86 ±11	0.8
Minimum systolic pressure (mmHg), mean (SD)	99 ±12	98 ±13	>0.9
Maximum systolic pressure (mmHg), mean (SD)	157 ±20	157 ±19	0.7
Bleeding (mL), median (IQR)	300 (100, 400)	300 (200, 400)	0.2
Urine (mL), median (IQR)	500 (300, 1,000)	600 (300, 1,000)	0.058
Fluid output (mL), median (IQR)	900 (488, 1,300)	900 (600, 1,400)	0.090
Fluid intake (mL), median (IQR)	2,558 (2,050, 3,065)	2,564 (2,072, 3,250)	0.4
Comorbidities, n (%)			
Hypertension	181 (44%)	72 (41%)	0.5
Diabetes	28 (6.9%)	9 (5.2%)	0.4
Laboratory indexes			
Creatinine (mmol/L), mean (SD)	62 ±19	62 ±19	0.7
Urea (mmol/L), mean (SD)	4.76 ±1.66	4.60 ±1.61	0.3
White blood cell (×109/L), mean (SD)	11.1 ±3.8	11.6 ±3.7	0.2
pH, mean (SD)	7.41 ±0.06	7.40 ±0.06	0.3
Bicarbonate (mmol/L), mean (SD)	23.1 ±2.8	22.4 ±2.8	0.009
Lactate (mmol/L), median (IQR)	2.00 (1.40, 2.90)	2.20 (1.50, 3.00)	0.067
Oxyhemoglobin saturation (%), median (IQR)	98.90 (97.70, 99.60)	98.95 (97.43, 99.60)	>0.9
Calcium (mmol/L), mean (SD)	2.03 ±0.14	2.03 ±0.14	>0.9
Albumin (g/L), mean (SD)	33.5 ±4.1	33.6 ±4.4	0.7
ICU length of stay (days), median (IQR)	6 (3, 12)	5 (3, 11)	0.8
Hosp. LOS (days), median (IQR)	19 (14, 24)	17 (12, 24)	0.14

Hospital mortality, n (%)	74 (18%)	38 (22%)	0.3

APACHE, Acute Physiology and Chronic Health Evaluation; RRT, renal replacement therapy; ICU, intensive care unit; AKI, acute kidney injury; Hosp. LOS, length of stay in hospital; IQR, interquartile range; SD, standard deviation.

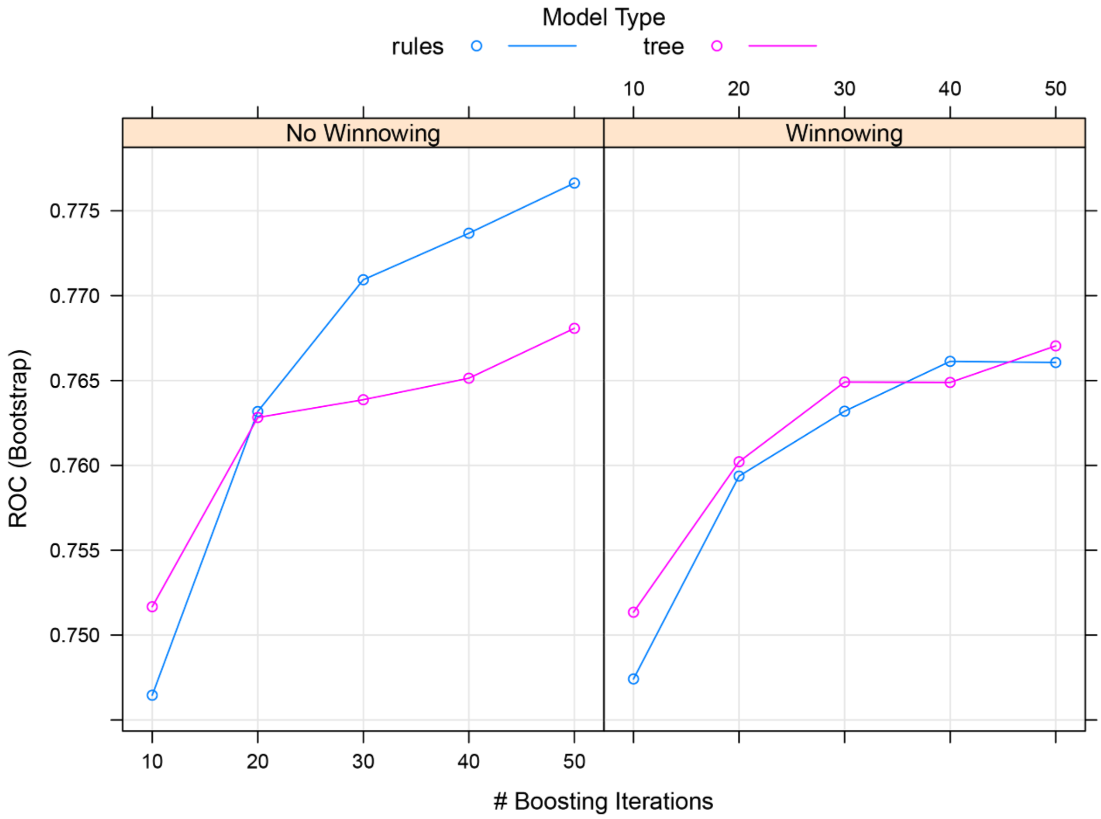

Figure S1. Hyperparameter tuning for the C5.0 machine-learning algorithm. Abbreviations: ROC, receiver operating characteristic.

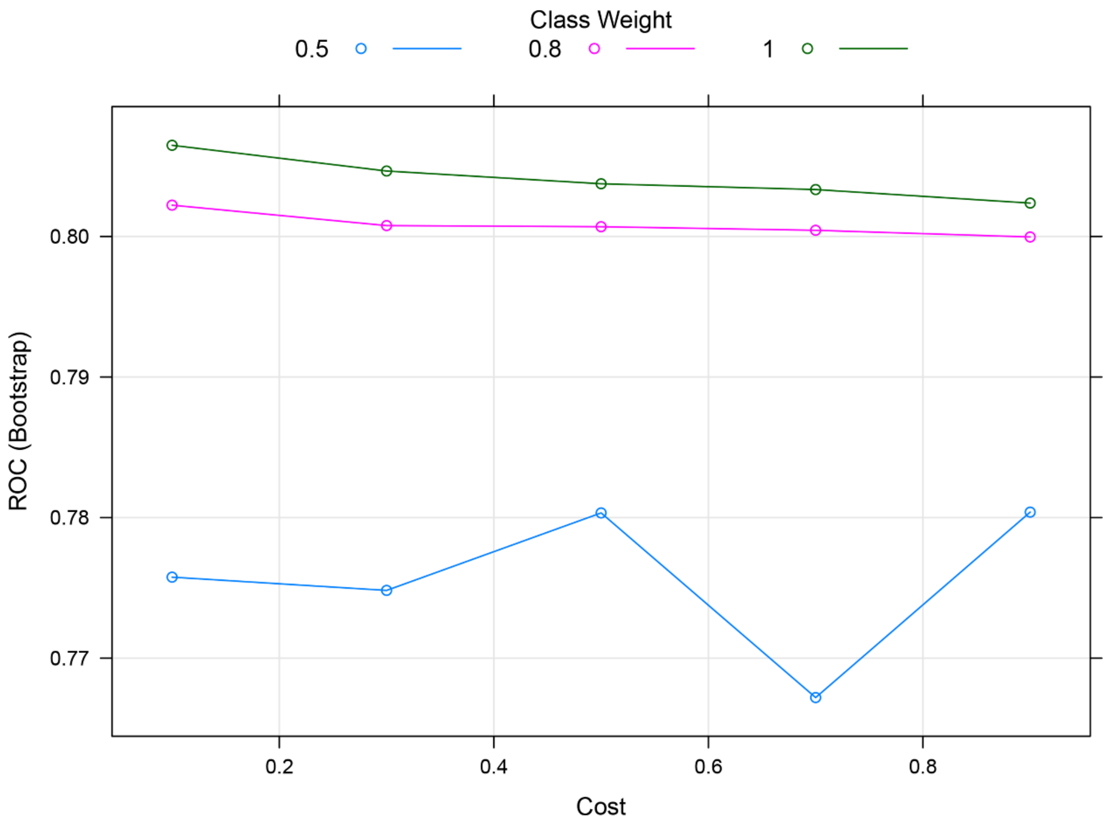

Figure S2. Hyperparameter tuning for the support vector machine machine-learning algorithm. Abbreviations: ROC, receiver operating characteristic.

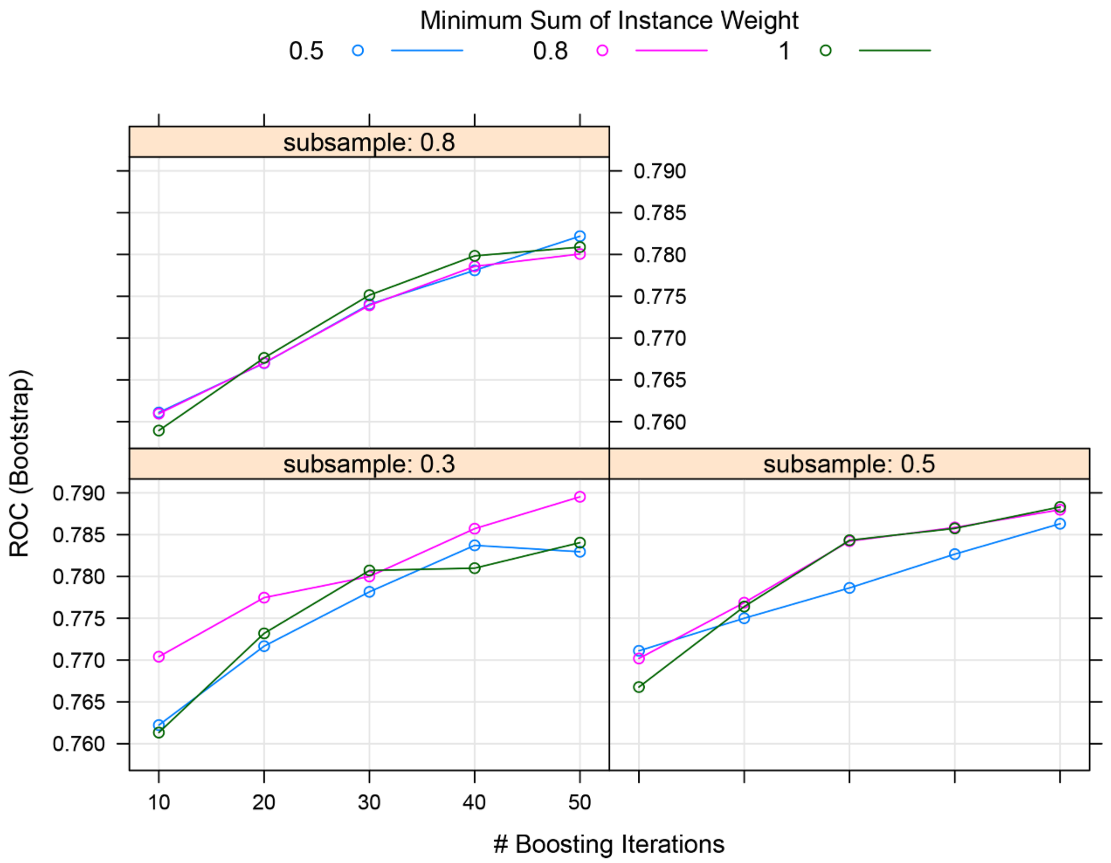

Figure S3. Hyperparameter tuning for the extreme gradient boosting machine-learning algorithm. Abbreviations: ROC, receiver operating characteristic.

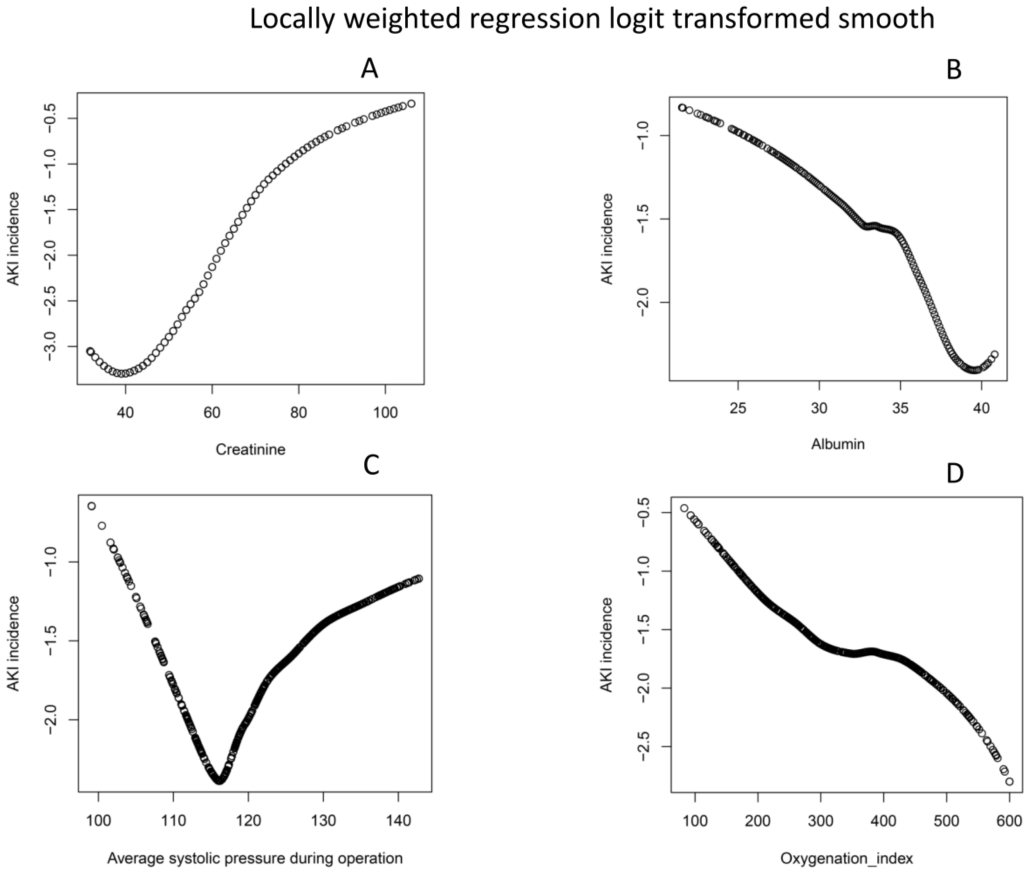

Figure S4. Crude relationship between top 4 features and acute kidney injury

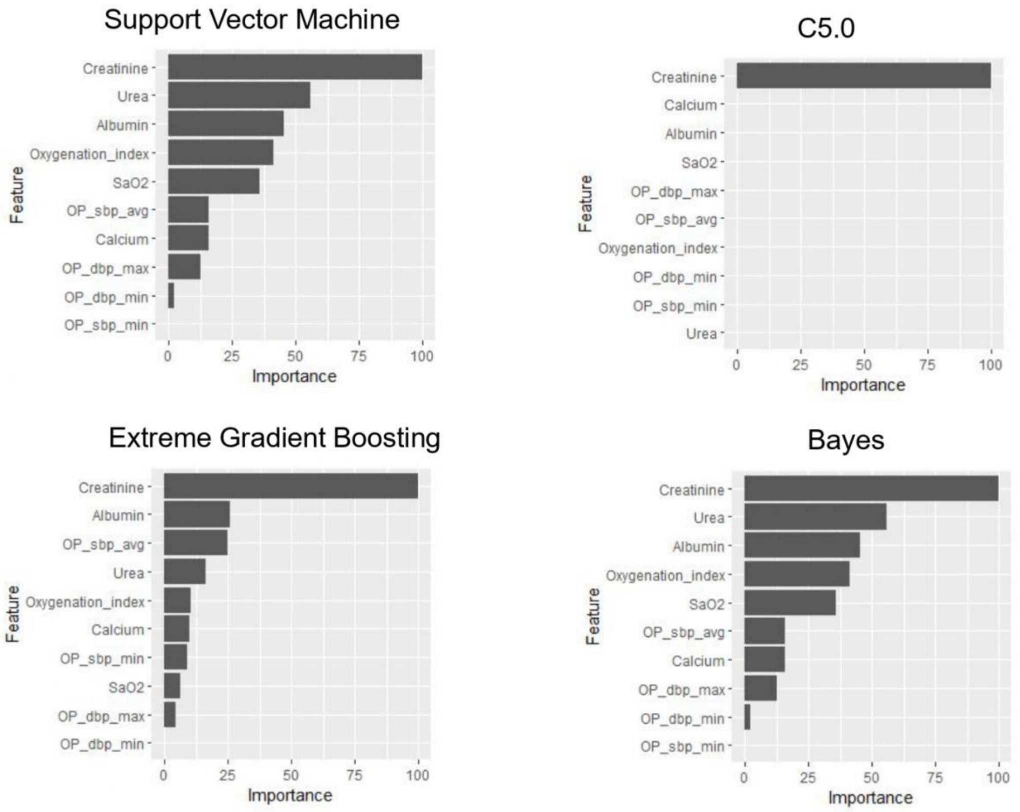

Figure S5. Variable-importance ranking in the four machine learning models

Abbreviations: SaO2, Oxyhemoglobin saturation; OP_sbp_avg, average systolic pressure; OP_dbp_min, Minimum diastolic pressure; OP_dbp_max, Maximum diastolic pressure; OP_sbp_min, Minimum systolic pressure.
